# Evolving Antibody Therapies for the Treatment of Type 1 Diabetes

**DOI:** 10.3389/fimmu.2020.624568

**Published:** 2021-02-18

**Authors:** Qi Ke, Charles J. Kroger, Matthew Clark, Roland M. Tisch

**Affiliations:** ^1^ Department of Microbiology and Immunology, University of North Carolina at Chapel Hill, Chapel Hill, NC, United States; ^2^ Lineberger Comprehensive Cancer Center, University of North Carolina at Chapel Hill, Chapel Hill, NC, United States

**Keywords:** diabetes, immunotherapy, monoclonal antibodies, immunoregulation, self-tolerance

## Abstract

Type 1 diabetes (T1D) is widely considered to be a T cell driven autoimmune disease resulting in reduced insulin production due to dysfunction/destruction of pancreatic β cells. Currently, there continues to be a need for immunotherapies that selectively reestablish persistent β cell-specific self-tolerance for the prevention and remission of T1D in the clinic. The utilization of monoclonal antibodies (mAb) is one strategy to target specific immune cell populations inducing autoimmune-driven pathology. Several mAb have proven to be clinically safe and exhibit varying degrees of efficacy in modulating autoimmunity, including T1D. Traditionally, mAb therapies have been used to deplete a targeted cell population regardless of antigenic specificity. However, this treatment strategy can prove detrimental resulting in the loss of acquired protective immunity. Nondepleting mAb have also been applied to modulate the function of immune effector cells. Recent studies have begun to define novel mechanisms associated with mAb-based immunotherapy that alter the function of targeted effector cell pools. These results suggest short course mAb therapies may have persistent effects for regaining and maintaining self-tolerance. Furthermore, the flexibility to manipulate mAb properties permits the development of novel strategies to target multiple antigens and/or deliver therapeutic drugs by a single mAb molecule. Here, we discuss current and potential future therapeutic mAb treatment strategies for T1D, and T cell-mediated autoimmunity.

## Introduction

Type 1 diabetes (T1D) is an autoimmune disease defined by the immune-mediated destruction and/or dysfunction of the insulin producing β cells within the pancreatic islets of Langerhans (1–11). Both genetic and ill-defined environmental factors (e.g. viral infection, diet) influence T1D susceptibility (4–6, 12–16). Typically, it takes a number of years from the initiation of autoimmunity to diagnosis of clinical diabetes (5–9). When the functional β cell mass is reduced by ~80%, production of insulin becomes insufficient to regulate the body’s glucose levels. Currently there is no established curative treatment, and T1D is managed *via* daily exogenous insulin treatment and monitoring of blood glucose levels. Insufficient control of daily glucose levels can lead to severe complications including blindness, atherosclerosis, and neuropathy (6, 7).

T1D is a consequence of the breakdown of peripheral tolerance to β cell antigens, such as proinsulin, insulin, and glutamic acid decarboxylase (GAD65). The triggering event of T1D is poorly understood, and likely involves an environmental insult. CD4^+^ and CD8^+^ T cells are generally considered to be the primary drivers of β cell destruction in T1D patients. For instance, the strongest genetic risk factor for T1D is associated with specific alleles of HLA class II and class I molecules, and CD4^+^ and CD8^+^ T cells are found infiltrating the islets of T1D subjects (5, 6, 9, 13–33). Furthermore, the more aggressive childhood versus adult T1D onset is marked by an expanded effector T cell (Teff) response to proinsulin and insulin (20–22). However, examples of human islets lacking a T cell infiltrate have also been reported (24, 34, 35). Other adaptive immune cell populations such a B cells, and various innate effectors such as dendritic cells (DC), macrophages (MΦ), and natural killer (NK) cells reside in the islets of T1D subjects as well (24, 34, 35). Autoantibodies to islet proteins are also detected prior to clinical T1D diagnosis, and have been used to establish the risk of individuals progressing to overt diabetes (36–41).

**Figure 1:**
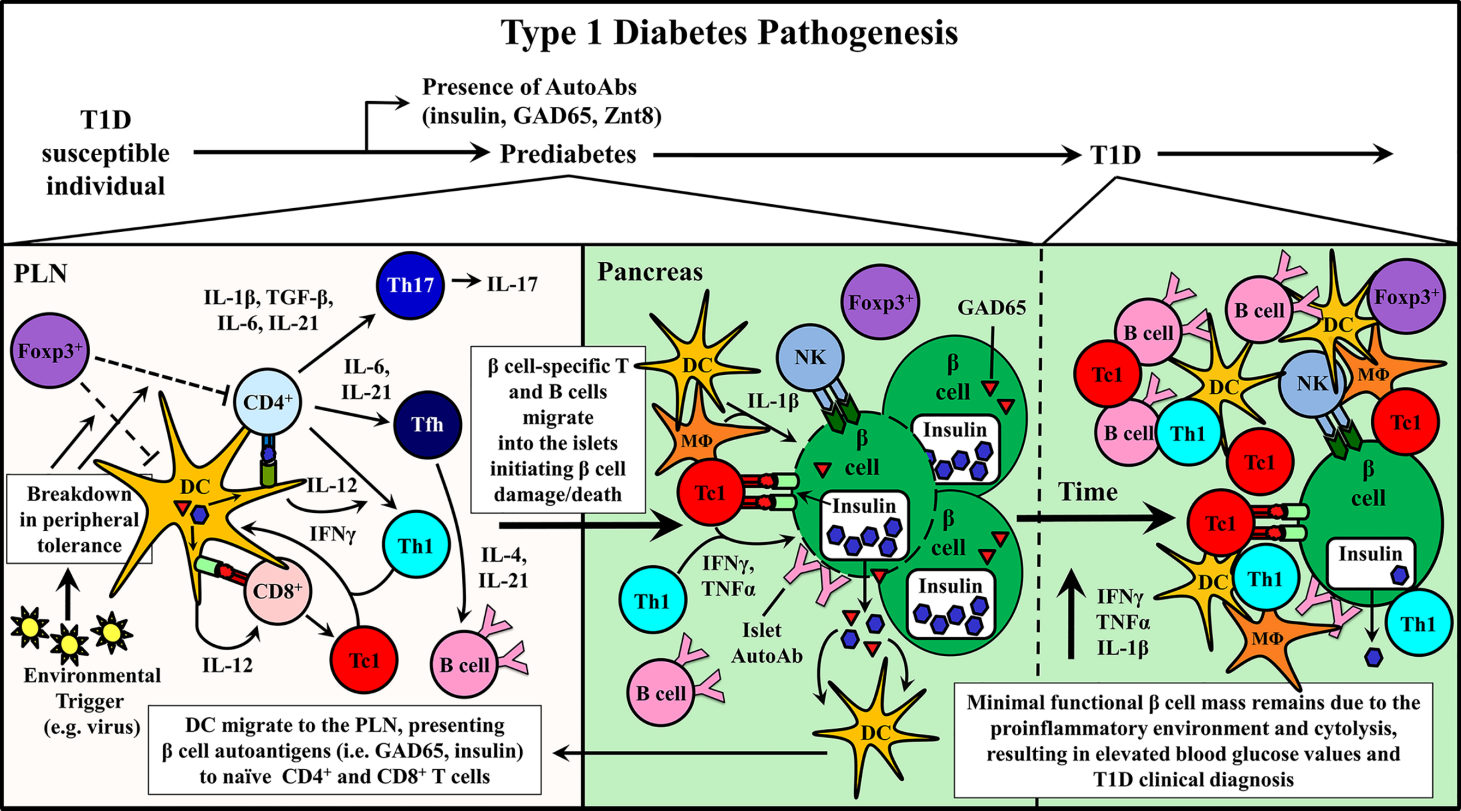
Type 1 diabetes (T1D) pathogenesis. Cellular events associated with driving T cell-mediated T1D are depicted within the pancreatic lymph node (PLN) and pancreas. Upon initiation of β cell autoimmunity *via* an ill-defined event, dendritic cells (DC) migrate from the pancreas ferrying islet autoantigens into the PLN. Here, naïve β cell-specific CD4^+^ and CD8^+^ T cells are activated and differentiate into distinct Teff subsets associated with T1D progression including CD8^+^ Tc1, CD4^+^ Th1, Th17, and Tfh. Early indication of ongoing autoimmunity is marked by the detection islet-specific autoantibodies (AutoAbs). Teff traffic into the pancreas and initiate β cell damage, which gradually increases over time prompting nominal insulin production.

Studies using the non-obese diabetic mouse (NOD), a model of spontaneous T1D have provided important information regarding disease progression and prevention (10, 11). Genetically manipulated NOD mice and adoptive transfer strategies have shown a direct role for CD4^+^ and CD8^+^ T cells as well as B cells in mediating β cell destruction. For example, in the absence of T or B cells, overt diabetes fails to develop (10, 11, 42–44). β cell-specific T cell reactivity is initiated by DC that ferry islet antigens from the pancreas into the draining pancreatic lymph node (PLN) (**Figure 1**) (45–49). In the PLN, naïve CD4^+^ and CD8^+^ T cells preferentially differentiate into proinflammatory Teff subsets, based on the cytokine *milieu* (**Figure 1**) (50–55). Release of IL-12 by DC induces the generation of type 1 CD4^+^ and CD8^+^ Teff, Th1 and Tc1, respectively, marked by expression of the transcription factor T-bet and the cytokine IFNγ (52, 56). Th1 and Tc1 cells have been closely linked to T1D development in both NOD mice and T1D patients (20, 52, 57, 58). However, IL-17A and IL-21-secreting Th17 cells, and IL-21-secreting T follicular helper (Tfh) cells also contribute to β cell destruction (50–52, 59–61). Th17 differentiation is driven by an IL-1β, IL-6, TGFβ, and IL-23 cytokine *milieu* (50, 52, 62), whereas IL-6 and IL-21 favor Tfh differentiation (51, 53–55). After APC-antigen encounter, self-reactive Teff migrate into the islets and promote β cell damage *via* direct cytolysis, and indirectly through production of proinflammatory cytokines, such as IFNγ, IL-1β and TNFα (**Figure 1**) (63–65). β cell damage and induced stress further exposes autoantigens, which leads to epitope spread and expansion of the pool of β cell-specific T cells (66, 67). Islet resident DC, MΦ and NK cells further promote β cell damage by maintaining the proinflammatory environment (5, 6, 9, 11, 24, 34, 45, 46, 57, 68–70). As islet inflammation or insulitis progresses, functional β cell mass declines until insulin production can no longer be sustained at sufficient levels to maintain appropriate blood glucose levels and overt diabetes is diagnosed (**Figure 1**) (5, 8, 9).

Based on findings made in NOD mice and T1D patients, T cells, and to a lesser extent B cells, have been the focus of most immunotherapy strategies (10, 11, 42–44, 71). Nevertheless, due to the heterogeneity and complexity inherent with the diabetogenic response, designing effective immunotherapies to prevent and/or treat T1D has been challenging. Numerous therapeutic strategies to prevent and/or reverse T1D have been met with varying degrees of clinical success and disappointment (72).

The use of monoclonal antibodies (mAb) has been one approach clinically tested to prevent and/or treat T1D and other autoimmune diseases (73–75). The development of therapeutic mAb involves a number of key steps including: mAb generation, screening/selection, humanization, affinity maturation, molecule optimization, and engineering for commercial production (73, 74). Notably, advances in *in vivo* and *in vitro* generation of antigen-specific mAb, and engineering of immunoglobulin (Ig) molecules have greatly aided the production and application of mAb for therapeutic use. Clinically applied mAb and related molecules have provided safe and selective therapeutic targeting of biologically relevant proteins for the treatment of several diseases ranging from cancer to autoimmunity (73–75). For instance, mAb therapy targeting TNFα is being used in rheumatoid arthritis (RA) to mitigate disease severity (76, 77).

**Figure 2:**
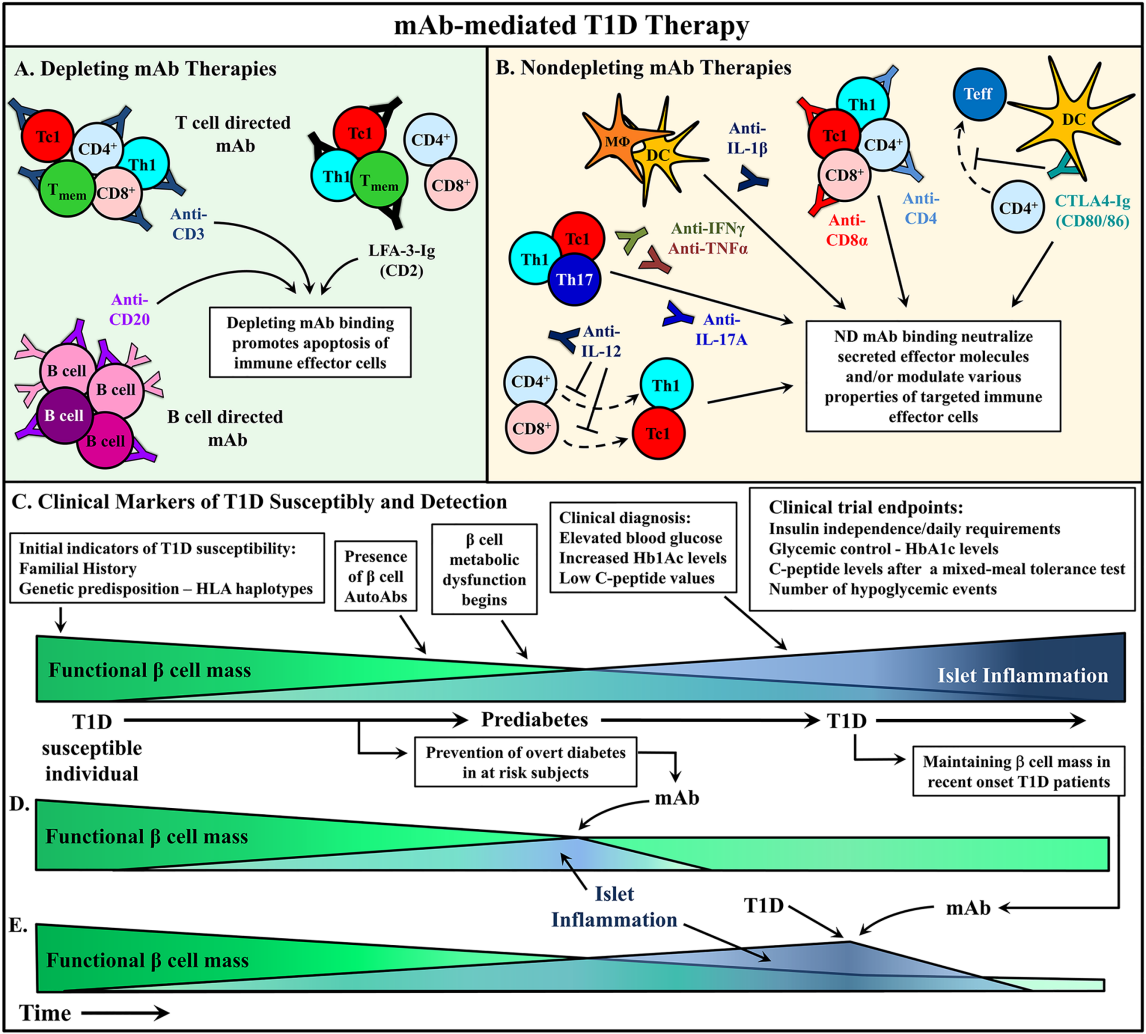
mAb therapies to ameliorate type 1 diabetes (T1D). **(A, B)** Monoclonal antibodies (mAb) treatments can be broadly divided into two categories: depleting mAb or nondepleting (ND)/neutralizing mAb. Some promising mAb treatments are depicted that have been used in either animal models or clinical trials to alter T1D progression. **(A)** Depleting mAb have been used to target T and B cells in the clinic. Transient depletion of T and B cells delays the progression of β cell autoimmunity. **(B)** ND mAb have been applied to neutralize cytokines to suppress the proinflammatory *milieu* of the pancreatic lymph node (PLN) and islets, as well as modulate the properties and activity of various immune effector cells. **(C)** The relationship between functional β cell mass versus islet inflammation is characterized. Over time, increased chronic islet inflammation results in decreased functional β cell mass, first detected *via* metabolic abnormalities, and ultimately leading to deficient insulin production, prompting clinical diagnosis of T1D. Individuals at different stages of T1D progression have treated with mAb therapies to alter T1D progression, and in turn **(D)** prevent diabetes onset, or **(E)** rescue residual β cell mass after clinical T1D diagnosis. Typically, these clinical trials have used metabolic readouts for β cell function as primary endpoints to determine therapeutic efficacy **(C)**.

In T1D, mAb treatment must suppress ongoing β cell destruction while reestablishing long-term self-tolerance. Maintenance of long-lasting self-tolerance is largely mediated by various subsets of regulatory T cells (Treg). The timing of T1D immunotherapy is believed to be a critical factor impacting clinical efficacy. Intervention with mAb at early stages of β cell autoimmunity, when the frequency of pathogenic immune effectors infiltrating the islets is relatively low and the functional β cell mass high, is expected to be the most effective time to modulate the autoimmune response. Alternatively, if treatment is started later, it may be necessary to couple mAb therapy with strategies that enhance the expansion and function of the residual β cell mass in recent onset and long-standing diabetic individuals. Therapeutic mAb typically function *via* two general mechanisms: i) depletion of target cell populations, and ii) blockade of cell receptor function (**Figure 2**). However, advancements in mAb development have provided novel uses for therapeutic mAb such as inducing select receptor signaling and the delivery of therapeutic drugs to a target cell. This review will discuss strategies applied and advancements made in mAb therapies for T1D prevention and treatment.

## Development of Therapeutic mAb

### Production of Antigen-Specific mAb

The advent of B cell hybridoma technology in the mid 1970’s provided the means to generate antigen-specific mAb, and in turn jump-started the field of mAb immunotherapy (78). The approach entails harvesting Ab-producing B cells from antigen immunized mice that are immortalized *via* fusion with myeloma cells to generate hybridoma cell lines (79). Although still a standard protocol for mAb production, the hybridoma method is generally time consuming and labor intensive (80). Accordingly, a variety of other approaches have been developed to provide more rapid production and expand the repertoire of antigen-specific mAb.

One such technique is phage display pioneered in the mid 1980’s (81). The general approach entails cloning a gene into gene III of filamentous phage, and having the encoded protein/peptide displayed on the surface of the phage (82). The engineered phages are then exposed to a protein that binds the ectopic protein/peptide, the protein-bound phages expanded in bacteria, and subjected to additional cycles of screening. This method has been adapted for Ab phage display (APD) to screen libraries of variable regions of antigen binding fragments (Fab) or recombinant single-chain variable fragments (scFv) expressed on the surface of phages (83). With large human Ig libraries readily available, the process of generating and screening sequences of complementary-determining regions (CDR) of human Ig is rapid (84). In addition, human Ig sequences negate the need for humanization (see below). Adalimumab (D2E7) was the first fully human anti-TNFα mAb developed using APD technology. Adalimumab exhibited comparable inhibitory efficiency to a murine anti-human TNFα mAb (MAK195), which was used as a template (85). APD can also be used for optimization of mAb generation and production (86, 87). This method allows immunization steps to be bypassed, which is a significant advantage for developing mAb against non-immunogenic, toxic, or self-antigens. Despite numerous benefits, a key drawback of APD is that the selection of heavy and light chains is based on random selection events that may not represent a functional Ig *in vivo* (88, 89). Nevertheless, APD provides an accelerated mAb discovery and screening method compared to the classic hybridoma mAb technique.

### Humanization of mAb

A murine mAb targeting the CD3 epsilon polypeptide of the human T cell receptor (TCR) complex was the first developed and approved for treatment in patients as an immunosuppressant drug to prevent acute allograft rejection after organ transplantation (90). Despite observed therapeutic benefits, severe side-effects emerged that limited clinical application (91). Patients treated with the murine mAb rapidly developed a human anti-mouse Ab (HAMA) response that ranged from the development of rashes to lethal kidney failure. In addition to significant safety issues, immune reactivity also reduces mAb efficacy and half-life. To overcome immune responses to mAb produced in non-human species, the Ig molecules undergo a “humanization” process. Here, non-human portions of the Ig molecule are reduced to minimize immunogenicity without compromising antigen binding. Initially, chimeric Ig molecules were generated consisting of a murine variable region coupled with a human constant region. However, the murine portion, making up ~30% of the Ig molecule, is still sufficient to elicit immune reactivity (92–94). Further mAb humanization, increasing the human content to ~85%, is accomplished by grafting the non-human CDR into similar human frameworks. However, this grafting can lead to the loss of antigen-binding affinity due to conformational alteration of the CDR loops (95, 96). The first FDA-approved humanized mAb daclizumab, an anti-CD25 mAb, was generated by grafting murine CDR, which resulted in markedly reduced binding affinity (97). Distinct residues in framework regions, known as vernier zone residues, are responsible for maintaining Ig binding affinity and need to be retained during humanization (98–100). However, murine residues found in vernier zones still can elicit HAMA responses (101). Therefore, efforts continue to preserve antigen binding while limiting HAMA responses for mAb engineered with a non-human binding domain on a human Ab backbone.

The use of transgenic rodents that express human (Hu)-Ig is one approach to generate *bona fida* human mAb following antigen immunization (102, 103). Transgenic mice, lacking endogenous Ig expression, have been established that express human light chain genes coupled with a germline human Vκ region. Human heavy chain genes encoding μ and γ1 are also expressed to allow class switching. Transgenic Hu-Ig mice have been used in combination with conventional hybridoma technology to produce several human mAb applied in the clinic including: zanolimumab (anti-CD4), canakinumab (anti-IL-1β), ustekinumab (anti-IL-12/23p40), and golimumab (anti-TNFα) (104–108).

### Fc Engineering

In addition to epitope binding, mAb elicit a wide range of effector functions that are dependent on the Ig Fc region. Effector functions include Ab-dependent cell-mediated cytotoxicity (ADCC), Ab-dependent cell-mediated phagocytosis (ADCP), and complement-dependent cytotoxicity (CDC) (109). Glycosylation of the Fc region impacts binding to Fc receptors (FcR) on effector cells and subsequent ADCC and ADCP responses. Accordingly, mAb effector function can be manipulated *via* modification of glycosylation of the Fc region. For example, enhanced ADCC by tumor resident NK cells is seen with a human IgG1 lacking fucosylated glycan at Asn297 in the Fc region, leading to increased tumor rejection (110, 111). Modification of the Fc region has also been important in reducing unwanted adverse events associated with mAb effector function. Initial clinical trials using anti-human CD3 OKT3 IgG1 for the treatment of T1D resulted in cytokine release syndrome (CRS), driven by FcRγ binding (112, 113). Alanine substitutions at amino acid positions 234 and 235 were introduced into the CH2 Fc region of the γ1 backbone that reduce glycosylation and FcRγ binding. Consequently, the resulting mAb, teplizumab (huOKT3γ1(Ala-Ala)), exhibits only minimal CRS (114). A similar approach has been used with the anti-human CD3 otelixizumab, a humanized aglycosylated IgG1 tested in T1D clinical trials (115, 116). The Fc region, however, is important for structural stability and reduced Fc binding to the neonatal receptor, FcRn, leads to shortened Ab serum half-life (117, 118). Therefore, engineering of the Fc region is important for mAb development that needs to be optimized for both drug safety and pharmacokinetics.

## The Application of Depleting mAb for T1D

mAb targeting cellular antigens are typically depleting due to ADCC, ADCP, and CDC responses. The goal of using a depleting mAb in the context of autoimmunity is to eliminate the pathogenic immune effectors preventing further tissue damage. In T1D, depleting mAb treatments in preclinical or clinical settings have targeted various cell populations. Transient depletion of T and B cells *via* mAb for example, have shown at least short-term benefits in recent onset T1D subjects (**Figure 2**) (119–123).

### Anti-CD3 Therapy

Arguably, the most successful clinical immunotherapy for T1D to date has been administration of anti-CD3 mAb. The first murine IgG2a specific for the human CD3 epsilon-subunit (OKT3) was developed in 1979, and approved by FDA as the first human mAb immunotherapy in 1986 (124). Treatment successfully prevented acute graft rejection and graft-versus-host-disease (GvHD) in organ transplant patients (124, 125).

In 1994, Chatenoud and Bach showed that anti-CD3 therapy reversed new onset diabetes in NOD mice, and established long-term remission and β-cell-specific tolerance (126). The mechanisms of protection induced by anti-CD3 mAb therapy have been extensively studied in mice (127–129). Following anti-CD3 mAb binding, increased TCR signaling promotes T cell activation-induced cell death (AICD) (130, 131). Interestingly, AICD by anti-CD3 mAb is selective for conventional T (Tconv) cells with limited effects on FoxP3-expressing CD4^+^ Treg (Foxp3^+^Treg) (132). In NOD mice, the depletion of islet infiltrating Teff by anti-CD3 suppresses ongoing β cell destruction, albeit at the expense of transient systemic depletion of Tconv (126, 133). Additionally, the ingestion of apoptotic T cells enhances TGFβ production by MΦ, which promotes Foxp3^+^Treg differentiation (128, 129, 134). This increased pool of Foxp3^+^Treg plays a critical role in maintenance of diabetes remission in NOD mice (128).

During a randomized, controlled, open-label phase I/II clinical trial, newly diagnosed T1D patients were given a 14-day course of treatment of teplizumab (120). Although diabetes reversal was not observed, the teplizumab-treated group had several promising metrics. Over a 2 year period C-peptide responses and insulin production were sustained, which correlated with decreased acetylated hemoglobin (HbA1c) and insulin dependency (119). Otelixizumab also was shown to preserve β cell function and reduce insulin use for 4 years in a phase II placebo-controlled trial (135). The beneficial effects of otelixizumab were most pronounced in patients with higher residual β cell function, but the therapeutic effects diminished by 24 months, suggesting overall efficacy was limited. In addition, otelixizumab treatment resulted reactivation of Epstein Barr virus in some subjects (121). Although the effects of otelixizumab were transient, this study indicated that intervening at an earlier time post-diagnosis enhanced efficacy.

Patients who received anti-CD3 mAb experienced significant reduction of peripheral T cells, which rebounded within a month after therapy (119, 120, 123, 136, 137). This reduction in numbers is in part believed to be due to T cell egress from the circulation (138). Evidence also indicated that anti-CD3 affected the T cell phenotype in treated T1D subjects. For example, the frequency of circulating central memory CD8^+^ T cells and exhausted islet-specific CD8^+^ T cells (TIGIT^+^KLRG1^+^PD-1^+^) were increased (136, 139, 140). Interestingly, recent studies show that aggressive T1D correlates with the presence of activated islet-specific HELIOS^+^ CD8^+^ memory T cells (Tmem) found in peripheral blood (141). On the other hand, slower progressing T1D is marked by peripheral blood islet-specific CD8^+^ Tmem exhibiting an exhausted phenotype characterized by upregulation of EOMES, 2B4, PD-1, TIGIT, and CD160 (141). These results suggest that anti-CD3 induced T cell exhaustion plays a role in the protective effect.

The phase III trials using either teplizumab or otelixizumab did not meet the primary endpoint goal (142–144). Nevertheless, *post-hoc* analyses showed a reduced loss of C-peptide in a subset of patients receiving teplizumab (142). On the other hand, the otelixizumab phase III trial was terminated after an untested and reduced dose of the mAb failed to significantly improve C-peptide levels compared to the control groups. A phase I/IIa repeat dose escalation study of otelixizumab has shown a dose-dependent relationship between anti-CD3-TCR engagement and TCR downregulation [NCT02000817 (145)]. The dosing of otelixizumab was also found to be well tolerated and preserved β cell function over 18 months (146). A new randomized, double-blind, placebo-controlled teplizumab phase III trial addresses the safety and tolerability of the treatment in recent-onset T1D young patients as well as the effect on β cell preservation using the most effective dosage and modified primary outcome based on earlier findings (NCT03875729).

A recent phase II study in high-risk, nondiabetic relatives of T1D patients, investigated the efficacy of teplizumab to prevent diabetes onset (NCT01030861). Subjects receiving a single 14-day course of teplizumab exhibited an average delay of 24 months in the onset of diabetes (136). Notably, the largest response to teplizumab was seen in subjects with reduced median levels of C peptide at the time of intervention, indicative of a later stage of disease progression. In addition, HLA haplotype was found to influence the efficacy of teplizumab. This latter result suggests that the TCR repertoire, likely reflecting the size of the activated Teff pool, are parameters influencing the response to teplizumab. Importantly, this study provides the first evidence that anti-CD3 treatment can delay T1D onset in at-risk individuals, as well as further substantiate targeting the T cell compartment as a general means to modulate the human disease process. Consequently, teplizumab has been granted PRIority MEdicines (PRIME) designation by European Medicines Agency and Breakthrough therapy designation by the U.S. Food and Drug Administration (147, 148).

The route of administration of anti-CD3 is also being assessed to enhance efficacy and safety. Initial studies with oral anti-CD3 treatment in murine experimental autoimmune encephalitis have demonstrated decreased side effects and increased efficacy at lower dosages (149). Of note, oral administration of anti-CD3 failed to alter the CD3/TCR complex or induce pronounced downstream TCR related signaling events such as depletion or release of proinflammatory cytokines (150). Instead tolerance was achieved by induction of TGFβ1-expressing Th3 cells (149, 151). However, the therapeutic efficacy of oral anti-CD3 treatment in T1D has yet to be clinically tested.

### Anti-CD20 Therapy

B cells are critical to the pathogenesis of T1D, and are consistently detected within the pancreatic islet infiltrate (9, 24, 42–44, 70). Development of diabetes is prevented in NOD mice treated with a B cell depleting anti-CD20 at a preclinical stage of T1D (**Figure 2**) (152). Similarly, anti-CD22 mediated B cell depletion in NOD mice prevents diabetes, and has been reported to induce remission in new onset animals (153).

Studies suggest that B cells are also key drivers in the progression of human T1D. For example, the aggressive, early onset of diabetes correlates with high numbers of islet infiltrating CD20-expressing B cells (57, 154). Rituximab, a mouse-human chimeric IgG1 mAb specific for human CD20, has been studied in recent onset T1D patients (122). One year after treatment, rituximab versus control treated subjects exhibited an improvement in the levels of HbA1c and C-peptide, as well as the requirement for insulin indicating preserved β cell function. However, CD19^+^ B cells slowly repopulated the periphery and no long-term benefit was detected after two years (155). Previous studies have indicated that disease promoting autoreactive B cells may not be completely deleted after anti-CD20 treatment (156, 157). Further studies regarding the timing, potential as a preventative treatment, and dosage of rituximab are needed to optimize this therapeutic potential for T1D.

### Anti-CD2 Therapy

CD2 is a surface adhesion molecule expressed by a variety of cell populations including T cells, NK cells and DC (158–160). Notably, CD2 levels are increased on Tmem (158, 159). Alefacept, a fusion protein consisting of the CD2-binding domain of LFA3 fused to the Fc region of human IgG1 (IgG1_Fc_), has been utilized to treat psoriasis (88, 161). The fusion protein preferentially binds to Tmem, and induces apoptosis mediated by IgG1_Fc_ binding to FcRγ expressed by NK cells (162). Accordingly, individuals treated with alefacept have reduced levels of Tmem. Regarding T1D, a 12 and 24 month clinical trial with recent onset patients demonstrated a reduced frequency of activated T cells and increased ratio of Treg to Tmem in blood. Additionally, C-peptide levels were improved after a mixed meal test (MMT) in the alefacept versus placebo group [NCT00965458 (163, 164)]. This correlated with reduced exogenous insulin requirements, and suggested that alefacept prolongs β cell function (163, 164). Interestingly, in psoriasis patients, alefacept reduced activated CD11c^+^/CD83^+^ DC subsets and inflammatory gene expression levels (165). Therefore, this strategy may restore peripheral tolerance *via* targeted deletion of activated self-reactive T cells and APC plus increasing Treg to dampen the ongoing autoimmune response.

Overall, depletion of immune populations *via* mAb has been effective in influencing β cell autoimmunity in both mice and humans. Nevertheless, there is the risk of limiting protective immunity following broad depletion of a given immune cell type, particularly if treatment requires continued mAb administration.

## The Application of Nondepleting mAb for T1D

In addition to depletion, mAb have the capacity to block and/or modulate intercellular and effector molecule interactions. Naturally occurring or engineered nondepleting (ND) mAb have been clinically used to block cytokine/chemokine-receptor interactions and to inhibit cell surface receptor-ligand engagement to affect an immune response (**Figure 2**). Human IgG4 for instance, has a low affinity for most FcRγ, which limits ADCC, ADCP, and CDC (166, 167). As noted above, modifying glycosylation patterns can also be used to block the depleting function of mAb, as well as enhance therapeutic efficacy, and Ig half-life (168, 169). Overall, ND mAb have the therapeutic advantage of preserving the pool of targeted immune effector cells while disrupting an ongoing autoimmune response.

### mAb Neutralization of Soluble Immune Effector Molecules

The combination of the proinflammatory cytokines IL-1β, IFNγ, and TNFα is cytotoxic to β cells (63, 64). IFNα also enhances CD8^+^ T cell-mediated destruction of β cells *via* upregulation of MHC class I by β cells (170–173). Therefore, mAb therapies have been used to neutralize the proinflammatory environment within the islets and preserve functional β cell mass (**Figure 2**). Two different therapies to inhibit IL-1β, an anti-IL-β mAb (canakinumab) and an antagonist of the IL-1R (anakinra), have been ineffective at maintaining β cell function in recent onset T1D patients [NCT00947427, NCT00711503 (174)]. In contrast, etanercept, an anti-TNFα fusion protein that binds to and removes TNFα from circulation, has demonstrated efficacy based on reduced Hb1Ac levels in recently diagnosed children [NCT00730392 (175)]. An ongoing clinical phase II trial is testing the tolerability and effects on β cell autoimmunity of etanercept in combination with vitamin D plus GAD65 prepared in Alum adjuvant (NCT02464033). The goal here is to suppress islet inflammation while inducing GAD65-specific Treg. Simponi, a neutralizing Ab that binds to both soluble and membrane bound TNFα, is also being investigated to maintain β cell mass (NCT02846545). Early evidence indicates that simponi may prolong insulin production, but long-term efficacy still needs to be determined (176).

Studies in NOD mice have shown that blocking IFNα or its receptor reduces T1D incidence (177, 178). Furthermore, mAb neutralization of IFNγ can prevent the progression of β cell autoimmunity in adoptive transfer models of T1D when administered at distinct treatment windows (179–181). In contrast, IFNγ-deficient NOD mice continue to develop diabetes, suggesting potential redundancy among proinflammatory cytokines and highlighting the importance how timing of mAb intervention can impact therapeutic efficacy (182–184). Overall, neutralizing a single proinflammatory cytokine has generally had limited success. This may in part reflect the relative role of a given cytokine in general and/or at a particular stage in the disease process. Targeting multiple cytokines that affect β cell viability and function may be needed to enhance the efficacy of the approach.

mAb targeting of cytokines to modulate T cell subset differentiation and effector function has also been a strategy to alter the progression of β cell autoimmunity. Neutralizing IL-12, which induces type 1 (e.g. Th1/Tc1) subset differentiation, limits insulitis and prevents the onset of diabetes in NOD mice (10). However, efficacy is dependent on continuous and frequent anti-IL-12 administration (185).

Cytokine-specific mAb have also been used to block the function and/or differentiation of other CD4^+^ T cell subsets involved in β cell autoimmunity, such as Th17 and Tfh cells (50, 52, 54, 55, 186). For instance, IL-21 secreted by Th17 and Tfh cells, has been targeted. A role for IL-21 in T1D was initially demonstrated in NOD mice lacking IL-21R, which exhibit minimal insulitis and reduced diabetes incidence (60). Ectopic expression of IL-21 by β cells also induces diabetes in non-autoimmune prone C57BL/6 mice (187). Furthermore, blockade of IL-21 reverses established T1D in NOD mice making IL-21 a promising therapeutic candidate in the treatment of T1D (188). Indeed, a phase II clinical trial of newly diagnosed T1D patients treated with anti-IL-21 and liraglutide (a glucagon-like-peptide-1 agonist), designed to curtail autoimmunity and boost insulin production, is underway (NCT02443155).

Diabetes incidence is reduced in NOD mice treated with anti-IL-17A starting at 10 weeks of age, a relatively late preclinical stage of T1D (59). Interestingly, protection correlates with an increased frequency of Foxp3^+^Treg in the islets and PLN, likely mediated in part by a dampened proinflammatory *milieu*. Both Foxp3^+^Treg and Th17 cells differentiate in the presence of TGFβ, however in the absence of additional proinflammatory cytokines such as IL-1β and IL-6, CD4^+^ T cell differentiation is skewed toward the Foxp3^+^Treg subset (189, 190). Notably, anti-IL-17A treatment initiated at an earlier stage of T1D progression is ineffective at preventing diabetes onset in NOD mice (59). These results suggest that interfering with Th17 differentiation and function is effective when insulitis is well established. Similarly, treatment of NOD mice at 10 weeks of age with recombinant IL-25, which antagonizes Th17 differentiation, also decreases T1D incidence (59). Thus, mAb therapies targeting Th17 and Tfh subsets have yielded promising results in murine T1D that may lead to beneficial clinical outcomes for human T1D treatment.

### Modulating Immune Effector Cell Activity *via* ND mAb

In addition to targeting soluble mediators regulating autoreactive Teff differentiation and function, ND mAb therapies have been applied to directly modulate Teff activity *via* binding to surface molecules. Naïve T cells have the plasticity to differentiate into various Teff subsets defined by unique transcription factor and cytokine profiles. One key factor driving Th1/Tc1 subset differentiation is a strong TCR signal, defined as the culmination of TCR (signal 1), co-stimulatory molecule (signal 2) and cytokine (signal 3) signaling pathways (191–193). Furthermore, continued TCR signaling is required to maintain Teff function. Therefore, strategies to dampen these signaling pathways are expected to prevent expansion and function of pathogenic Teff. Blocking the T cell co-stimulatory molecule pathway using abatacept, an anti-CTLA4-Ig fusion protein, slows β cell functional decline and improves Hb1Ac values in new-onset T1D patients, although insulin independence is not achieved [NCT00505375 (194, 195)]. Interestingly, recent studies have indicated clinical responsiveness to abatacept in recent-onset T1D subjects is dependent on suppression of Tfh cells, indicating that the therapy modulates the T cell pool (61).

Short-term ND mAb treatment targeting the T cell co-receptors CD4 and CD8α both prevents and reverses diabetes in NOD mice while establishing long-term β cell-specific tolerance (196–200). T cells examined shortly after mAb treatment exhibit reduced TCR signaling and suppressed production of proinflammatory cytokines (196, 197). Importantly, mAb-bound T cells rapidly egress from the islets and PLN (196–198). The latter is dependent on mAb-mediated co-receptor cross-linking, and is marked by distinct changes in T cell transcriptional activity, decreased sensitivity to local retention cues, and enhanced responsiveness to tissue egress-inducing chemokines (196, 197). Long-term maintenance of tolerance is tissue-specific, mediated by an induced β cell-specific Foxp3^+^Treg, while protective immunity is unperturbed (198). Interestingly, a ND humanized anti-human CD4 IgG1 mAb, tregalizumab, has been reported to preferentially activate and enhance the suppressor activity of FOXP3^+^Treg *in vitro* (201). Short-term ND mAb treatment strategies targeting T cell co-stimulatory molecules can inhibit β cell-specific T cell reactivity long-term *via* changes in T cell transcriptional profiles.

mAb that recognize β cell-peptide-MHC complexes may provide an additional strategy to alter TCR signaling, and enhance targeting of autoreactive Teff. For example, a mAb (mAb287) recognizing the peptide:MHC class II complex insulin B:9-23 peptide in the context of IA^g7^ blocks IL-2 cytokine secretion and tetramer binding by an insulin specific T cell hybridoma. Additionally, NOD mice treated weekly starting at 4 weeks of age with mAb287 significantly delays T1D onset (202).

mAb-mediated blockade of CD127, the IL-7Rα, both prevents and reverses diabetes in NOD mice (203, 204). CD127 is expressed by naïve T cells and Tmem, and is critical for maintaining T cell homeostasis. Short-term anti-CD127 treatment induces increased PD-1 expression and diminished proinflammatory cytokine production by Teff, consistent with an exhausted T cell phenotype (203, 204). Indeed, the protective effect induced by anti-CD127 mAb in NOD mice is reversed by treatment with a PD-1 blocking mAb, known to rescue exhausted T cells (203). T1D patients treated with the anti-IL-7Rα mAb RN168 show a reduction in Tmem and activated T cells while the FOXP3^+^Treg pool is maintained (205). Nevertheless, C-peptide levels are not markedly altered, which may reflect the dose and/or duration of RN168 administered.

ND mAb therapy has also been employed to alter NK cell activity. Specifically, studies have examined the role of NK cell activating receptor NKp46 or the mouse orthologue NCR1 in affecting the diabetogenic response (68, 206, 207). Anti-NCR1 mAb (NCR1.15) treatment initiated early in disease progression decreased diabetes incidence in NOD mice (206). This protective effect correlated with a pool of NK cells with reduced NCR1 surface expression, activation and degranulation (206). Although the ligand for NKp46 has yet to be identified, NCR1-Ig and NKp46-Ig fusion proteins bind to murine and human β cells respectively, indicating that β cells express a NK cell activating ligand (68, 208). Recently a humanized anti-NKp46 (hNKp46.02) has been shown to also reduce NK cell degranulation and internalization of the NKp46 activating ligand (207). Therefore, future studies are poised to investigate the efficacy of targeting NK cells for T1D treatment.

Central to the development of T1D is the ability of pathogenic autoreactive immune cells to traffic into the islets (209). T and B cells require various adhesion molecules to facilitate extravasation from circulation into sites of inflammation. Notably, NOD mice deficient in the T cell adhesion molecule ICAM-1 are protected from diabetes (209, 210). Accordingly, mAb-mediated blockade of adhesion molecules has been assessed in NOD mice as a means to prevent T and B cell trafficking into the islets (209). NOD mice treated with anti-ICAM-1 mAb exhibit reduced diabetes onset (209, 211). Similarly, blockade of MADCAM-1 in young NOD mice also reduces diabetes incidence. However, once islet infiltration has been established, MADCAM-1 blockade is ineffective (209, 212). These findings suggest that the timing of mAb blockade of adhesion molecules is critical, and that the approach is more effective at earlier stages of β cell autoimmunity when only a limited number if islets are infiltrated.

Taken together, ND mAb offer a promising therapeutic approach for prevention and treatment of T1D. Initial clinical and a substantial number of preclinical studies demonstrate that the function of effector molecules and properties of various immune cell types driving β cell autoimmunity can be modulated by ND mAb and fusion molecules. This is achieved without significant changes in systemic numbers of immune effectors or disruption of protective immunity.

### mAb-Cytokine Complexes

Cytokine-based therapy has been used to modulate immune-mediated pathology, including autoimmunity and T1D. However, systemic delivery of cytokines is problematic due to pleiotropic effects, and non-specific cell signaling that leads to potentially severe adverse effects. To overcome these obstacles, mAb-cytokine complexes are being developed and applied to target specific cell populations, and in this way enhance efficacy and safety. An example is the use of IL-2-Ab complexes (213).

IL-2 is predominantly produced by activated T cells and promotes expansion and survival of Teff (214). Additionally, IL-2 is essential for Foxp3^+^Treg differentiation, fitness, and maintenance (214). Development of murine T1D has been linked to reduced IL-2 production by T cells leading to Foxp3^+^Treg dysfunction and heightened β-cell-specific Teff responses (215–221). Similarly, polymorphisms in the *CD25* gene are associated with human T1D susceptibility, and in part, result in reduced sensitivity of FOXP3^+^Treg to IL-2 (215–222). Upon activation the IL-2 receptor complex, consisting of CD25, CD122, and CD132, is upregulated on Tconv. In contrast, Foxp3^+^Treg constitutively express elevated levels of the high affinity IL-2 receptor component CD25 (223). Increased CD25 expression by Foxp3^+^Treg provides a competitive advantage to acquire local IL-2 and therefore prevent Teff expansion and function (221, 223). Two murine anti-IL-2 mAb have been developed that exhibit distinct biological functions *in vivo* when bound to recombinant IL-2 (213, 224). The anti-IL-2 clone S4B6 establishes an IL-2 complex that preferentially binds to Teff. S4B6 binding to IL-2 prevents IL-2 interaction with CD25 causing selective binding of IL-2 to CD122 on Teff (213, 224). In contrast, the JES-61A2 anti-IL-2 clone establishes an IL-2 complex that is preferentially bound by Foxp3^+^Treg (213, 224). Here JES-61A2 binding to IL-2 prevents interaction with CD122 causing IL-2 to signal through CD25 (213, 224). Co-treatment of NOD mice with β cell peptide loaded tetramers and IL-2-JES-61A2 mAb complexes selectively expands β cell-specific Foxp3^+^Treg reducing diabetes incidence (225). A key benefit of mAb-IL-2 complexes is that the half-life of IL-2 is extended which aids pharmacokinetics (226). Low dosage IgG-IL-2 complexes significantly enhances FOXP3^+^Treg numbers and function in human peripheral blood as well as in cynomolgus monkeys for the treatment of GvHD (227).

### Bispecific mAb

Bispecific Ab (bsAb) contain two distinct antigen binding sites. Structurally bsAb typically consist of two different Fab arms, or two unique Ab linked by a common Fc region (228). bsAb can be used in autoimmunity to: i) neutralize multiple cytokines or receptors simultaneously, ii) force cell-cell interactions of different immune populations, and iii) initiate receptor co-localization on the cell surface (228, 229). The first clinically applied bsAb was blinatumomab, a CD19- and CD3-specific recombinant, for the treatment of non-Hodgkins B cell lymphoma. Blinatumomab forces an interaction between B cells and cytotoxic T cells. The result of this interaction is efficient elimination of the B lymphoma cells, expansion of protective T cells, and an increased life expectancy in the majority of patients (228, 230).

A bsAb specific for the β cell specific glucose transporter 2 molecule (Glut2) and the T cell inhibitory receptor CTLA-4 has been tested in NOD mice (231). This bsAb binds to β cells *via* Glut2 and engages CTLA-4 on Teff to suppress function. Glut2-CTLA-4-specific bsAb treatment of NOD mice results in reduced T cell proliferation and proinflammatory cytokine production, and decreased diabetes incidence (231). While the application of bsAb for the treatment of T1D has been limited to date, several enticing therapeutic strategies exist. bsAb that promote interactions between Foxp3^+^Treg and Teff, such as a CD25 and CD122, would be one approach. Another therapeutic option would be to use bsAb to establish “dual” anti-inflammatory cytokine complexes. An IL-12-IL-2 mAb fusion protein for example, has been used to simultaneously deliver both IL-2 and IL-12 to enhance Teff and NK function for cancer treatment (232). In the context of autoimmunity, dual cytokine fusion complexes of IL-2 and TGFβ may prove to be an effective strategy to induce and expand adaptive Foxp3^+^Treg. As autoimmune diseases are driven by several events, bsAb provide a novel therapeutic avenue to modulate multiple drivers of autoimmunity simultaneously.

## Summary

The ultimate goal of an immunotherapy for T1D is to suppress ongoing β cell autoimmunity by restoring peripheral tolerance without affecting protective immunity, and preserve β cell function. The complexity of the disease process, marked by multiple immune effectors (**Figure 1**), and varying kinetics of disease progression among individuals, however, has made the development of effective immunotherapies highly challenging to date (5–8, 72).

In general, mAb therapies have been applied to alter disease progression by deleting immune effector cells, altering effector cell phenotype/function or blocking soluble/cell-surface protein interactions (**Figure 2**). Clinical therapies targeting T and B cells *via* anti-CD3 and anti-CD20, respectively, have demonstrated safety and efficacy in maintaining β cell mass in newly-diagnosed patients (**Figure 2**) (136, 233, 234). However, these therapies and others fail to reestablish self-tolerance long-term. This may in part be due to insufficient induction/expansion of FOXP3^+^Treg or adaptive Treg subsets, and/or failure to adequately tolerize relevant pools of pathogenic Teff and Tmem. In this regard, it is noteworthy that efficacy of anti-CD3 to delay diabetes onset in at risk subjects is in part dependent on HLA haplotype (136). This finding suggests that parameters such as TCR repertoire, the avidity/affinity of the Teff, and/or the size of the pathogenic Teff/Tmem pool contribute to therapeutic outcome. The results are intriguing and further underscore the complexity associated with effectively manipulating the complete autoimmune response.

One key variable that influences the efficacy of mAb in T1D is the timing of intervention in relationship to disease progression (7, 8, 72). The diabetogenic response in human T1D can be viewed as a succession of stages marked by: 1) the initiation of autoimmunity detected by presentation of multiple islet autoantibodies, 2) ongoing autoimmunity with the presentation of metabolic abnormalities that indicate aberrant stress on β cell mass, and 3) the onset of overt diabetes indicating loss of function of the majority of β cell mass (**Figure 2**). The majority of clinical trial interventions have been applied at the second and third stages of disease progression. It is well established by preclinical studies that a given mAb treatment may only be effective at a particular stage of T1D progression (72). Recent clinical results indicating that the efficacy of anti-CD3 therapy to delay diabetes onset is dependent on ongoing autoimmunity, further highlight this key aspect of T1D immunotherapy. Notably, a similar temporal effect is seen in NOD mice in which anti-CD3 therapy fails to prevent diabetes onset when given to NOD mice at an early stage in disease progression (133). The nature of the effector cells and molecules being targeted will ultimately determine clinical efficacy at a given stage of T1D progression. Here, it is critical that the mechanism by which a therapeutic mAb induces tolerance be fully understood to help better predict efficacy when administered at a given stage of disease progression, as well as the likelihood of induction of long-term tolerance without the need of persistent intervention. At earlier and less stringent stages of T1D, strategies that limit trafficking of effectors into the islets and/or activation and/or differentiation of pathogenic effectors are expected to be effective. In contrast, during late preclinical T1D stages or at the onset of diabetes the therapy must be sufficiently robust to rapidly tolerize an established and sizable pool of islet resident pathogenic effectors. In both settings induction and/or expansion of Treg subsets is needed to maintain tolerance by limiting subsequent differentiation, expansion and/or function of pathogenic Teff. In an attempt to minimize temporal effects of disease progression, mAb therapies that establish long-term, β cell-specific tolerance broadly over preclinical and clinical T1D stages in NOD mice and other rodent autoimmune models, need to be identified and prioritized. In this regard, the use of ND mAb specific for CD4 and CD8 is noteworthy. A short course of ND anti-CD4 and CD8α mAb prevents diabetes onset when administered to young NOD mice and results in rapid reversal of diabetes and tissue-specific long-term tolerance in NOD mice (196–199, 235).

In view of the complexities of the diabetogenic response in general, and the varied parameters linked to effectively tolerizing immune effectors, it is likely that multiple cell types and/or effector molecules will need to be targeted with combinations of mAb (**Figure 1**). For example, expansion of β cell-specific Foxp3^+^Treg may be enhanced by combining mAb-IL-2 complexes with mAb that quench the proinflammatory *milieu* of the PLN and islets and/or block Teff differentiation and function (225). Alternatively, β cell-specific Treg subsets can be induced and/or expanded *via* more traditional antigen-specific based strategies following “broad” tolerization of Teff *via* mAb therapy. The application of bsAb may be particularly advantageous for combinatorial strategies. The simultaneous targeting of multiple proteins (e.g. proinflammatory cytokines) by a single therapeutic agent simplifies treatment dosing and regimen, and limits potential drug-drug interactions (228, 229). Importantly, regardless of the mAb strategy, the ability to restore lasting peripheral tolerance to prevent further β cell destruction is necessary for clinical success.

An important consideration in selecting a given set of mAb strategies is whether β cell autoimmunity is driven by T cells versus innate cells, and/or β cell intrinsic defects. Currently, evidence indicates that the rapid, aggressive disease developing in children is largely T cell-mediated. However, adult onset T1D may be driven by T cells, and/or innate effectors and/or β cell intrinsic defects leading to dysregulation of insulin production (236). Needed are sensitive disease readouts and biomarkers that distinguish between the respective scenarios or endotypes, to ensure that the appropriate therapeutic strategy is being applied (237). Consideration is also needed in defining successful endpoints for testing a therapeutic approach in the clinic (237). The ideal scenario is that a given mAb immunotherapy protects or rescues β cell function measured in part by metabolic indicators (**Figure 2**). Nevertheless, in the absence of a successful metabolic outcome, there is much value in determining whether an immunotherapy has induced tolerance within the targeted effector cell pool. As alluded to above, establishing tolerance in one compartment of the disease process may be insufficient to achieve a therapeutic benefit. However, determining that tolerance is indeed established would provide justification to combine in a rational manner, appropriate complementary strategies to enhance therapeutic efficacy. In the case of T cells, single cell transcriptome analysis of T cells bound by β cell-specific multimers and sorted from the blood of test and control subjects would be one approach sufficiently sensitive to detect changes within the T cell compartment.

To date, mAb therapies have provided intriguing results in affecting the progression of T1D. Nevertheless, an effective strategy to reestablish self-tolerance long-term is still required. Ongoing T1D research continues to characterize novel genes and potential targets involved in T1D disease susceptibility and progression. The ability to customize the mAb target and respective effector function provides immense flexibility to discover and develop a successful mAb treatment for T1D.

## Author Contributions

QK, CK, MC, and RT contributed to the preparation of the review article. All authors approved the submitted version.

## Funding

This work was supported by National Institutes of Health grants R01DK100256, R01AI139475, R01AI141631, R21AI115752 (RT), and T32AI007273 (MC), and the American Diabetes Association 1-18-PDF-108 (MC).

## Conflict of Interest

The authors declare that the research was conducted in the absence of any commercial or financial relationships that could be construed as a potential conflict of interest.
